# Genetic Regulation Mechanism of Cadmium Accumulation and Its Utilization in Rice Breeding

**DOI:** 10.3390/ijms24021247

**Published:** 2023-01-08

**Authors:** Guang Chen, Ruiying Du, Xu Wang

**Affiliations:** 1 Institute of Quality Standard and Monitoring Technology for Agro-Products of Guangdong Academy of Agricultural Sciences, Guangzhou 510640, China; 2 Key Laboratory of Testing and Evaluation for Agro-product Safety and Quality, Ministry of Agriculture and Rural Affairs, Guangzhou 510640, China; 3 Guangdong Provincial Key Laboratory of Quality & Safety Risk Assessment for Agro-Products, Guangzhou 510640, China

**Keywords:** rice, cadmium, absorption and transport, molecular mechanism, low-cadmium breeding

## Abstract

Cadmium (Cd) is a heavy metal whose pollution in rice fields leads to varying degrees of Cd accumulation in rice. Furthermore, the long-term consumption of Cd-contaminated rice is harmful to human health. Therefore, it is of great theoretical significance and application value to clarify the genetic regulation mechanism of Cd accumulation in rice and cultivate rice varieties with low Cd accumulation for the safe use of Cd-contaminated soils. This review summarizes the effects of Cd on rice growth, yield, and quality; the physiological and molecular mechanisms of Cd absorption in the roots, loading, and transport of Cd in the xylem, the distribution of Cd in nodes, redistribution of Cd in leaves, and accumulation of Cd in the grains; the regulation mechanism of the Cd stress response; and the breeding of rice with low Cd accumulation. Future directions on the genetic regulation of Cd in rice and application are also discussed. This review provides a theoretical basis for studies exploring the genetic regulation of Cd stress in rice. It also offers a basis for formulating effective strategies to reduce the Cd content in rice.

## 1. Introduction

Cadmium (Cd) is a non-essential element in plants. It naturally exists in nature under low concentrations; thus, it does not affect human health in a normal environment. However, with the development of the modern industry in the 20th century, Cd has been widely applied in many fields as a semiconductor, electroplating, feed, and fertilizer. At the same time, Cd pollution in the atmosphere and water has become increasingly serious, resulting in the accumulation of Cd in farmland. The use of livestock manure and mineral fertilizers have also increased Cd accumulation [1,2,3]. Cd is highly mobile and toxic, so it is easily enriched in the human body through the food chain.

Rice and its products are a staple food for people in southern China and most areas in Asia. In 2017, the total rice yield in China was 212.68 million tons, accounting for about 32% of the total grain production [4]. However, Cd is easily accumulated in rice grains, which increases the risk of exposure to Cd in Chinese people. The 2008 national survey on the rice market revealed that about 10% of rice had a Cd content exceeding the national food safety limit of 0.2 mg·kg^−1^ [5]. In addition, the Cd content in rice growing in areas in southern China where nonferrous metal is produced is exceedingly higher than the standard limit [6]. For example, in the northern Hunan Province, the Cd content in rice exceeds the standard by 60%, with the Cd content in 11% of the rice exceeding 1 mg·kg^−1^[6]. Furthermore, the Cd content in 76% of rice samples in the middle and east of Hunan exceeded the standard Cd limit [7]. Incidences of “Cd-polluted rice” in Hunan, Guangdong, and Jiangxi have been reported by the media since 2013, increasing the public concern about the safety of rice for human consumption. Therefore, identifying the factors influencing Cd accumulation in rice and proposing corresponding prevention and control measures is of great significance in ensuring food safety and human health.

Cd uptake can lead to cell damage, interfere with the normal physiological metabolism of cells, and affect plant growth and development. In order to resist Cd toxicity, plants have evolved a variety of detoxification mechanisms including limiting root Cd uptake, preventing Cd from entering cells, chelating, vacuolar storage, and activating antioxidant defense systems [8]. At present, two main strategies have been applied to control Cd contamination in rice. The first strategy aims at preventing the Cd in paddy soil from entering the plants through the roots using chemical and biological methods. The second strategy promotes the adoption of appropriate agronomic measures and farming systems to reduce the accumulation of Cd in rice. However, adopting the genetic breeding technology to breed low-Cd rice varieties is the most direct and effective way to ensure the safe production of rice [9]. This review discusses the effects of Cd stress on the growth, yield, and quality of rice, and the physiological and molecular mechanisms of Cd uptake and transport in rice. In addition, the potential strategies to reduce Cd accumulation in rice based on the latest progress in breeding low-Cd rice are presented. The present review provides a theoretical and practical basis for screening and breeding low-Cd rice.

## 2. Cd Stress Inhibits the Growth, Yield, and Quality of Rice

Cd toxicity has a negative impact on rice morphology, physiology, and biochemistry, which significantly hinders rice growth, leading to yield reduction and the deterioration of rice quality [10]. We will discuss these aspects in detail below.

### 2.1. Effects of Cd on Physiological Characteristics of Rice

Excessive accumulation of Cd in rice induces toxic effects, which reduce the content of photosynthetic pigments, respiratory intensity, transpiration, and photochemical efficiency of rice plants. Cd strongly inhibits the protochlorophyllide reductase activity and blocks the synthesis of aminol-γ-evulinic acid, affecting the biosynthesis of chlorophyll and its stable binding with protein. As a result, the transmission of photosynthetic electrons in photosystem I (PSI) and photosystem II (PSII) is destroyed, hindering photophosphorylation and the synthesis of ATP, and subsequently, photosynthesis in chloroplasts [11,12]. In this process, PSII is more sensitive to Cd than PSI [13]. The effect of Cd on photosynthesis also destroys the chlorophyll–protein complex by forming the Cd–chlorophyll complex [12]. For example, the chlorophyll content and the indices of the photosynthetic characteristics (net photosynthetic rate, stomatal conductance, transpiration rate, and intercellular CO_2_ concentration) in rice seedling leaves showed a downward trend with the increase in Cd concentration [10]. At the same time, the activity of amylase was significantly inhibited under high Cd stress, reducing starch hydrolysis, which inhibits root elongation due to the deficiency of the materials and energy required for radicle and hypocotyl growth [14]. In addition, the accumulation of Cd inhibited the activities of respiratory-related enzymes including malate dehydrogenase, succinate dehydrogenase, glucose-6-phosphate dehydrogenase, and 6-phosphate gluconate dehydrogenase in rice plant cells, resulting in plant respiratory metabolism disorder [15]. Meanwhile, many reactive oxygen species free radicals are produced in plants under Cd stress. The free radicals attack the unsaturated fatty acids on the cell membrane, loosening the cell membrane structure, which lowers the membrane function [16]. The increase in malondialdehyde content under Cd stress damages the membrane system, leading to a disorder in cell metabolism [17]. Cd stress also enhances the activities of important protective enzymes in the antioxidant system of rice such as superoxide dismutase, peroxidase, and catalase, which reduce oxidative stress damage on plants [18].

### 2.2. Effects of Cd on Rice Yield

Varying effects of cadmium stress on rice yield have been reported in different studies [9]. Huang et al. [19] and Li et al. [20] found that Cd-treated soil had little effect on the seed setting rate and 1000-grain weight of potted rice. Ding et al. [21] found that different Cd concentrations in soil had no significant effect on the 1000-grain weight and grain number per panicle of rice, but the economic yield of rice grown in high Cd soil decreased by 10.65%. However, Chen revealed that Cd treatment significantly lowered all indicators of rice yield, especially the number of grains per panicle relative to the control, implying that Cd stress mainly affects the rice yields by affecting the differentiation of young panicles [22]. Additionally, Liu et al. revealed that the number of grains per panicle and the rice grain weight decreased, with no significant change in the 1000-grain weight with increased Cd concentration [23]. Ge [24] found that Cd treatment had the most obvious inhibitory effect on the panicle number of rice, but had less of an inhibitory effect on the 1000-grain weight. These inconsistent results may be attributed to the differences in rice genotypes and Cd treatment methods. The effect of Cd on rice yield is mainly in the early growth stage by affecting the rice photosynthesis, respiration, and absorption of mineral elements, so that the normal development of rice is blocked, resulting in reduced rice production. In terms of yield reduction effect, hybrid rice is more sensitive to Cd pollution than inbred rice [25].

### 2.3. Effects of Cd on Rice Quality

According to the National Food Safety Standard (GB 2762-2017) [26], the Cd content in rice should be lower than 0.2 mg·kg^−1^. Cd accumulation in grains affects the appearance, processing, and nutritional quality of rice. Precisely, Cd-contaminated rice has high concentrations of gliadin, less concentration of albumin and globulin, and lower content of lysine and amylose; thus, it is nutritionally poor [19,27]. Furthermore, the disintegration and peak viscosity values of rice with a high concentration of Cd are significantly decreased, while the degradation value, final viscosity, and hot paste viscosity are significantly increased, implying that Cd reduces the taste quality of rice [19]. Li et al. also revealed that irrigating rice plants using water with a Cd concentration lower than 0.012 mg·L^−1^ significantly reduced the quality of the rice appearance, although it had no significant impact on the nutrient composition [20]. At the same time, the content of amylose and Cd in brown rice grown on Cd-polluted soils was significantly increased, while the protein content in rice grains was significantly reduced in rice under low-Cd polluted soils [21].

## 3. Physiological and Molecular Mechanisms of Cd Accumulation in Rice

**Figure 1:**
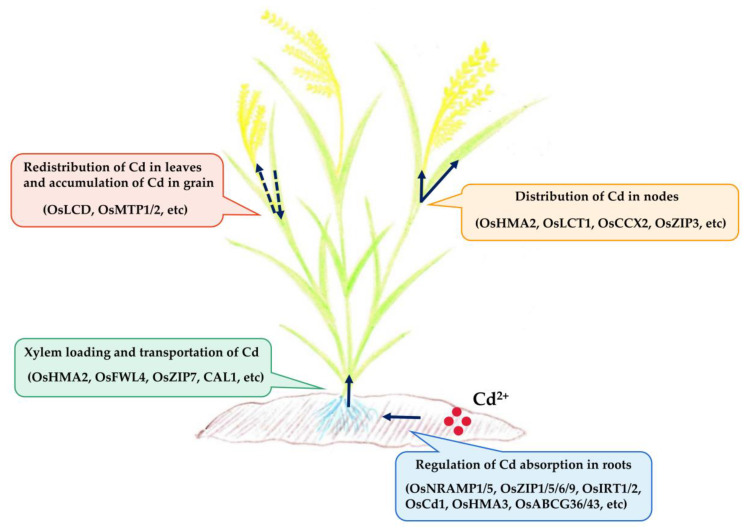
Schematic diagram of the Cd accumulation in rice.

Cd accumulation in rice is a complex physiological process involving the integrated functions of multiple cells, tissues, and organs (Figure 1). It mainly includes four processes: (1) Cd absorption in the roots; (2) loading and transportation of Cd in the xylem; (3) distribution of Cd in the nodes; and (4) the redistribution of Cd in the leaves and accumulation of Cd in the grains [28,29].

### 3.1. Cd Absorption by the Roots

Cd is absorbed from the soil through the rice roots. The Cd content in the soil changes with the soil pH and the organic matter concentration around the roots, and is mainly referred to as the effective Cd [30]. Effective Cd first comes into contact with the root epidermal cells. However, two layers of a protective-net-like structure exist outside the epidermis, which can effectively prevent Cd from flowing to the inner side of the rice roots. The first layer is an “iron plaque” consisting of iron and manganese, which is distributed in patches on the outside of the rice roots, and its thickness changes with the different growth stages of rice [31]. The iron plaque can competitively inhibit Cd, reducing the capacity of Cd absorption through the roots [32]. The other layer is the cell wall of the epidermal cells, which contains a large amount of cellulose, hemicellulose, pectin, and lignin [33]. These substances can chelate Cd, inhibiting its movement in the roots.

If the Cd passes through the iron plaque and cell wall, it moves into the inner root through two pathways [29]. The first is the apoplastic pathway. Through this pathway, the Cd ions passively move to the endodermis along the concentration gradient through the intercellular space [34]. However, the Cd transported via the apoplastic pathway is blocked by endodermis and exodermis, since the endodermis and exodermis in rice roots have Kjeldahl bands [29]. Instead, the Cd ions enter the cells of the endodermis and exodermis through transporters (Table 1) before they are further transported to the stele. Alternatively, the Cd ions are actively transported to the inner root structure through the symplast pathway, where Cd enters the cells through the transport proteins before moving to the symplast through the plasmodesmata.

*OsNRAMP5*, a member of the natural resistance-associated macrophage protein (NRAMP) family, is the main Cd transporter encoding gene for Cd transport through the rice root cells. It is expressed in the epidermis, exodermis, and tissues around the xylem in the roots. *OsNRAMP5*-encoded protein is localized in the cytoplasmic membrane, where it mediates the transport of manganese, Cd, and iron [36,56,57]. In rice, the OsNRAMP5 protein is polarly localized at the distal side of the exodermis and endodermis cells in the apical mature zone, which verifies the role of OsNRAMP5 in the absorption of heavy metals by the rice roots. Furthermore, the contents of manganese and Cd in the stems and grains of *OsNRAMP5*-knockout rice were significantly decreased compared to wild-type (WT) rice [56]. These findings are consistent with those of Yang et al. [58], who analyzed the T-DNA insertion mutant of *OsNRAMP5*. At the same time, *OsNRAMP5*-knockout rice with low Cd accumulation (*japonica* and *indica* rice) has been generated using CRISPR/Cas9 gene-editing technology, which clarifies the important role of *OsNRAMP5* protein in manganese and Cd absorption by rice [59].

Since the radius and extra-nuclear electron conformation of Cd ions are close to the multiple divalent cations essential in many plants, Cd, among other nutrient elements such as zinc (Zn) and iron, enter the cells through the common channel [60,61]. In addition, Tan et al. identified two ZIP family genes, *OsZIP5* and *OsZIP9*, as tandemly repeated genes, encoding Zn-regulated transporters (ZRTs), which play a coordinating role in Zn and Cd transport in rice [39]. *OsZIP5* and *OsZIP9* were expressed in the root epidermis, where they encoded the plasma membrane-localized proteins with absorption activity. At the same time, the Zn/Cd accumulation in the mutants *oszip5*, *oszip9*, and *oszip5*/*oszip9* was decreased. Plasma membrane-localized *OsZIP6* was also involved in the absorption of ions by the root cells. Using the patch clamp technique, verification in Xenopus laevis oocytes revealed that OsZIP6 substrates consist of six metal ions including cadmium, zinc, iron, cobalt, manganese, and nickel [40].

Cd can also enter the root cells with the help of Fe-transporters. For example, Fe transporters, OsIRT1 and OsIRT2 displayed a Cd ion transport activity in yeast. Under iron deficiency conditions, OsIRT1 facilitates iron and Cd absorption, while the OsIRT2 protein has a stronger transport activity and broader substrate specificity [43]. In addition, the Cd content in the roots and shoots of *OsIRT1*-overexpressed rice plants is higher than in the WT under high Cd culture conditions [62]. *OsNRAMP1* is significantly upregulated under iron deficiency. *OsNRAMP1* is localized on the plasma membrane, which participates in the absorption of Cd and iron through the roots. Furthermore, the expression level of *OsNRAMP1* in high-Cd accumulating varieties is higher than in the low-Cd accumulating varieties [35,63]. *OsCd1*, a member of the major facilitator superfamily (MFS), regulates Cd transport and is mainly expressed on the plasma membrane of rice roots. *OsCd1* mutation significantly reduces the efficiency of Cd absorption in rice and the amount of Cd accumulated in grains. Furthermore, the natural variation of *OsCd1* is the major determinant for the differences in Cd accumulation in grains of *indica* and *japonica* rice [44].

Following Cd absorption through the root cells, some Cd is accumulated into vacuoles, limiting its transport to the aboveground tissues and grains [46]. The *OsHMA3* transporter plays an important role in Cd entering vacuoles. *OsHMA3* belongs to the heavy metal ATPase (HMA) family and is located on the tonoplast membrane of root cells. It regulates the compartmentalization of Cd in the root vacuoles, which affects the transport rate of Cd from the rice roots to shoots [64]. The high-Cd accumulating rice variety Cho Ko Koku (CKK) has been proven to have a strong root-to-shoot transfer rate, controlled by a recessive allele (*OsHMA3mc*) for *qCdT7*. Since *OsHMA3mc* has lost its transport function, it does not mediate the transport of Cd into the vacuoles. Thus, most of the Cd in the root is loaded into the xylem, resulting in an increased transport rate of Cd to the shoot in CKK. Compared to the control plants, the growth of rice overexpressing *OsHMA3* is slightly inhibited under Cd stress, with an increased Cd concentration in the root but decreased concentration in the shoot [65]. Furthermore, seven nucleotide variations in the region between −683 and −557 bp of the OsHMA3 promoter showed obvious differentiation between *indica* and *japonica* rice, a key factor controlling the difference in Cd accumulation between *indica* and *japonica* rice [66].

Once absorbed into the root cells, some Cd is effluxed into the intercellular space, reducing the Cd damage to cells. The efflux of Cd or Cd chelated from the root cells is mediated by *OsABCG36*, which belongs to the ATP-binding cassette transporter superfamily (ABC), also known as OsPDR9. *OsABCG36* plays an important role in Cd tolerance in rice. Thus, Cd induces the expression of *OsABCG36* in the root tissues except for the epidermis since *OsABCG36* is located on the plasma membrane. As a result, the knockout of *OsABCG36* increases the Cd accumulation in root cells, which enhances the Cd sensitivity [47]. OsABCG43 (OsPDR5) also has a Cd-transport activity. The *OsABCG43* gene has been reported to improve Cd resistance in yeast [48]. OsABCG43 is expressed in the roots and shoots in rice, with Cd treatment inducing its expression in the roots, although the specific expression mechanism is unknown. 

OsZIP1 is a metal efflux transporter that limits the excessive accumulation of Zn, Cu, and Cd in rice, located on the plasma membrane and endoplasmic reticulum [37]. *OsZIP1* is abundantly expressed in the roots and is induced by excessive Cd accumulation at the transcription and translation levels. *OsZIP1*-overexpressing rice grows better under Cd stress, with less Cd accumulation in the rice plants, while the Cd accumulation in the roots of mutant *oszip1* and RNAi plants increases, implying that it is a significantly sensitive phenotype.

### 3.2. Loading and Transport of Cd in the Xylem

Part of the Cd absorbed by the roots is transported through the exodermis and endodermis into the xylem vessel. Once in the root xylem vessel, Cd is transported to the shoot through the loading, transport, and unloading of the xylem by root pressure and transpiration. This process is considered as an important transport link that mediates the Cd accumulation in the shoot. A comparison of the relationship between the xylem loading capacity and the Cd content in various aboveground parts of different rice varieties revealed that the Cd transport from the root to the shoot is the main physiological process determining the Cd accumulation level in grains [67]. Furthermore, the dynamic changes in Cd in high-Cd and low-Cd-accumulating rice varieties have been documented using the positron-emitting tracer imaging system [68]. Overall, the Cd transport potential to the shoots is stronger in high-Cd-accumulating rice varieties, implying that xylem transport plays an important role in Cd accumulation in rice.

The *OsHMA2* gene encodes a protein located on the plasma membrane, which transports Zn and Cd and is expressed in the root vascular bundle and phloem in the stem nodes at the heading and filling stage. The Zn and Cd concentrations in the leaves of the *OsHMA2*-mutant are decreased, while the Zn concentration in the roots is increased, implying that *OsHMA2* plays a role in Zn and Cd loading into the xylem. In addition, the Cd concentration in *OsHMA2*-overexpressing rice grains is reduced, which has potential application value for cultivating low-Cd rice [69]. 

The membrane protein family (MPF) containing the PLAC8 domain CCXXXXCPC or CLXXXXCPC and OsFWL4 is also involved in Cd transport in rice. In yeast, the expression of *OsFWL4* in yeast mutant *∆ycf1* enhances Cd resistance. Furthermore, Cd is transported from the root of *OsFWL4*-RNAi rice plants to the shoot, and the content of Cd in the shoot is significantly reduced, implying that *OsFWL4* acts as a transport protein, directly participating in the Cd transportation from the root to the shoot [49]. *OsZIP7*, encoding a plasma membrane-localized protein, is also expressed in the vascular bundle parenchyma cells in the roots and stem nodes, where it participates in the transport of Zn and Cd [41]. The knockout of *OsZIP7* in rice results in Cd retention in the roots and stem nodes, implying that it may be involved in the xylem loading in the roots and Cd transport between the vascular bundles in the stem nodes.

A quantitative trait locus *CAL1* (cadmium accumulation in leaf 1) has been identified in rice, which encodes a defensin-like protein rich in cysteine, with multiple thiol groups expressed in the root exodermis and xylem parenchyma cells [50,70]. CAL1 promotes the secretion of Cd into the extracellular space by chelating Cd in the cytoplasm, which reduces the concentration of Cd in the cytoplasm while promoting the long-distance transport of Cd through the xylem vessels. CAL1 specifically regulates the Cd transport from the rice root to the shoot, with no significant impact on the accumulation of Cd or other essential metal elements in the grains. Therefore, it can be used to cultivate new varieties with excessive Cd accumulating in the rice straw and the Cd in the grains not exceeding the standard. These new varieties can repair paddy soil and produce safe rice grains [50,70].

### 3.3. Cd Distribution in Stem Nodes

The stem node is the junction between the leaf and the stem and is an important site responsible for Cd transport from the root to the stem and spike [71]. In rice, the interconnected vascular bundles including (1) enlarged vascular bundle (EVB); (2) transit vascular bundle (TVB); and (3) diffuse vascular bundle (DVB), localized around the EVB, are distributed between the stem nodes. Multiple layers of parenchyma cells rich in plasmodesmata, referred to as the parenchyma cell bridge (PCB), are distributed between EVB and DVB [72,73]. Cd in the roots enters the EVB xylem with sap flow, and part of it is unloaded through the xylem into the DVB through PCB-mediated horizontal transport and DVB reloading. The other part of Cd enters the leaves, where it is reactivated, returning to the stem node through the phloem and into the DVB phloem through the sieve tube at the base of the stem node [72]. Considering that the phloem is the main channel for Cd deposition into the grains, and the DVB in Node I is directly connected with the rice panicle, the Cd transport from EVB to DVB through PCB or sieve tube at the base of stem node is of great significance in Cd entry into the grains [28].

*OsHMA2* is not only expressed in the roots but also in the phloem of stem nodes during the reproductive stage of rice. OsHMA2 plays a key role in the preferential transport of Zn and Cd from the rice stem nodes to developing tissues [45]. At the same time, *OsLCT1*, a low-affinity cation transporter (LCT) located on the plasma membrane, has a Cd efflux activity. *OsLCT1* is expressed in all developmental stages of rice, with a higher expression in the stem nodes and leaves at the reproductive stage. It is mainly accumulated in the cells around EVB and DVB in Node I. The inhibition of the *OsLCT1* expression via RNAi does not affect the Cd transport in the xylem tissues in the stem nodes; rather, the Cd content in the phloem sap is significantly reduced. At the same time, the Cd accumulated in the grain is about half that in the control plant, implying that OsLCT1 plays a role in the Cd transport through the stem node phloem to the grain [51]. 

Cation/Ca exchangers (CCXs) belong to the cation/calcium superfamily (CaCA). CaCA family members transport Ca^2+^ across the membrane in a reverse concentration gradient coupled with the transport of H^+^, K^+^, or Na^+^. Precisely, OsCCX2 participates in Cd accumulation in the grains through the Ca^2+^ transport pathway in rice stem nodes [52]. *OsCCX2* encodes a plasma membrane-localized efflux transporter, mainly expressed in the xylem tissues in the stem nodes. The knockout of *OsCCX2* significantly decreases the Cd content in the grains with a significantly increased Cd content in the roots and stem nodes, implying that OsCCX2 is involved in Cd transport between the xylem vessels in the stem nodes. At the same time, *OsZIP3*, which encodes a Zn transporter, is expressed in Node I in rice. It is responsible for unloading Zn from the EVB xylem [38]. OsZIP3 also transports Cd [74], but the specific mechanism for Cd accumulation in rice is still unclear.

### 3.4. Redistribution of Cd in Leaves and Cd Accumulation in Grains

Part of the Cd loaded into the xylem and transported to the stem nodes is accumulated in the rice panicles before heading, while the rest of the Cd is transported into the rice leaves [75]. The Cd content in the leaves is decreased during grain filling, while that in the stems and panicles increases, implying that Cd accumulated in the leaves is redistributed to other organs at maturity [76]. Thus, the Cd in the grains comes from the other organs during vegetative growth or is directly transported through the roots during reproductive growth [77]. In addition, the Cd concentration in rice phloem sap is the key determinant of the Cd content in grains [78].

*OsLCD* is a single-copy non-homologous gene, localized in the cytoplasm and nucleus, which is mainly expressed in the vascular tissues in the roots and the phloem companion cells in the leaves [53]. *OsLCD*-knockout inhibits the Cd transport to the shoot and grains, reducing the Cd content in the grains. A protein structure analysis revealed that the OsLCD protein is poor in cysteine, with no heavy metal detoxification function, transmembrane domains, and transport function. However, the OsLCD protein regulates the Cd accumulation and transport in rice by interacting with other proteins. Precisely, 21 proteins interacting with OsLCD have been identified using yeast two-hybrid technology including six proteins related to material transport and five proteins related to stress tolerance in plants [79].

Metal tolerance proteins (MTPs) belong to the cation diffusion facilitator (CDF) protein family [80]. Ten *MTP* genes have been identified in rice including *OsMTP1*, which belongs to the Zn-CDF subfamily. *OsMTP1* is mainly expressed in the vascular bundles in the leaves, where it effectively transports Cd while maintaining ion homeostasis in plants [81]. *OsMTP1* dsRNAi transgenic rice seedlings with a decreased tolerance to Cd have been generated [54]. Furthermore, in rice, *OsMTP2* has been identified as a membrane protein gene in the MTP family, which is mainly expressed in the leaves and stems, and plays an important role in metal ion transport during vegetative rice growth [55].

## 4. Regulatory Mechanism of the Cd Stress Response

Long non-coding RNAs (lncRNAs) and microRNAs (miRNAs) are key regulators of Cd stress responses in plant cells. According to Liang et al., lncRNAs play an important role in Cd stress response based on the whole-genome expression profile of lncRNAs induced by Cd in rice roots through deep sequencing [82]. Furthermore, RNA-sequencing of the roots of rice seedlings treated with Cd identified nine lncRNAs highly correlated with Cd-stress response [83]. At the same time, Ding et al. identified 19 miRNAs in response to Cd stress, of which 18 were downregulated, while miR528 was upregulated under Cd stress [84,85]. Subsequent research has revealed that Cd stress significantly induces the expression of miR268, but the expression of the target gene *OsNRAMP3* is significantly reduced. Precisely, the Cd content in miR268-overexpressing rice seedlings was higher than in the WT, implying that miR268 is a negative regulator of Cd tolerance in rice [85]. In addition, the miR390-overexpressing rice is more sensitive to Cd stress, accumulating higher Cd levels than in the WT, with increased expression of *OsHMA2* and *OsNRAMP5*, which implies that miR390 is also a negative regulator of rice tolerance to Cd stress [86]. 

The homeodomain-containing protein 4 (*OsHB4*) gene encodes the HD-Zip protein and participates in stress responses. The overexpression of miR166 inhibits the expression of *OsHB4*, which reduces Cd transport from the roots to the shoots, accumulates Cd in the grains, and alleviates Cd-induced oxidative stress [87]. In addition, the target genes of miR159 and miR167 encode the ABC transporter and NRAMP1b metal transporter under Cd stress, respectively [88,89]. The overexpression of miR192 inhibits the expression of the ABC transporter gene and negatively regulates rice seed germination under Cd stress [90]. At present, studies on lncRNAs and miRNAs are significantly limited, and the biological functions of most lncRNAs and miRNAs are still unclear. Therefore, it is necessary to pay more attention to this research field and improve our understanding of the regulatory mechanism of rice responses under Cd stress.

Transcription factors regulate the spatiotemporal expression of Cd-related genes in rice, determining the response of rice to Cd stress. For example, OsTTA is a plant transcription factor in the homeodomain-finger (PHD-finger) family, which regulates the transcription of metal transporter genes. In addition, in the *low cadmium 5* (*lc5*) rice mutant, the expression of *OsNRAMP1*, *OsZIP1*, and *OsIRT1*, and the concentration of various divalent metal ions including Cd are decreased [91]. NAC is another transcription factor belonging to the plant-specific transcription factor family, which enhances plant stress tolerance. OsNAC3 is a transcriptional regulator for Cd tolerance in rice, enhancing Cd tolerance of the Cd-sensitive yeast mutant *∆ycf1*. Furthermore, the Cd tolerance of the *OsNAC3* T-DNA insertion mutant *nac3* is decreased, implying that it is involved in response to Cd stress in rice [92]. At the same time, *OsNAC300* is expressed in the roots and is induced by Cd. Precisely, the Cd tolerance is enhanced in *OsNAC300* overexpression lines, while the tolerance is reduced in its mutant, implying that it plays a role in the rice tolerance to Cd stress [93]. 

OsHsfA4a is a heat shock transcription factor (Hsf) influencing Cd tolerance in rice. Precisely, the Cd tolerance of rice is decreased with the reduction in *OsHsfA4a* expression. OsHsfA4a also improves the Cd tolerance of rice by inducing the expression of metallothionein genes in the roots, although the specific mechanism is yet to be confirmed [94]. MYB is another typical transcription factor family in plants. In rice, OsMYB45 plays an important role in tolerance against Cd stress. It is highly expressed in rice leaves, glumes, stamens, pistils, and lateral roots under Cd stress. For example, the *osmyb45* mutant developed shorter plants and old necrotic leaves following Cd treatment, while the H_2_O_2_ content in the leaves increased, and the catalase activity decreased. OsMYB45 alleviates the damage caused by Cd on cells by scavenging the peroxide in cells, thereby improving the Cd tolerance in plants [95]. At the same time, the WRKY transcription factors contain one to two conserved domains, constituting a protein superfamily in higher plants [96]. The *OsWRKY15* is significantly induced by Cd, implying that it plays a role in the response and resistance of rice against Cd stress through the NO signaling pathway [97].

## 5. Breeding of Low-Cd Accumulation Rice

**Figure 2:**
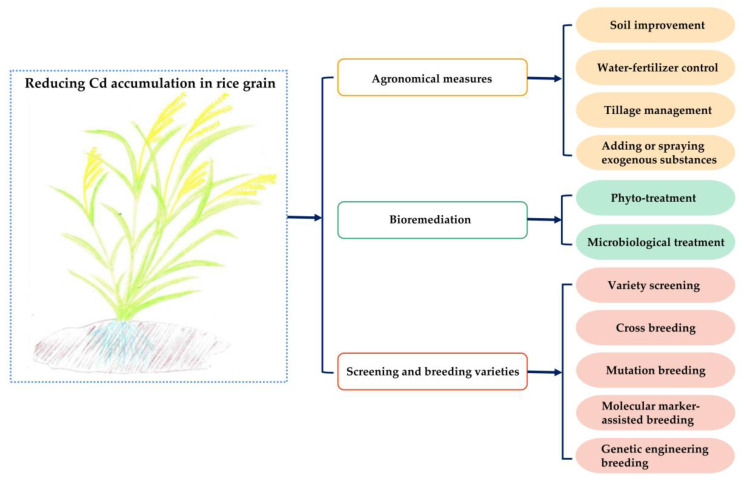
The main strategies for reducing Cd accumulation in rice grains.

Reducing Cd pollution in rice fields and decreasing Cd accumulation in rice grains has long been a research focus in agricultural science. At present, three strategies can be employed to reduce Cd accumulation in rice grains (Figure 2) [98]. These strategies include (1) Cd regulation through agronomical measures such as soil improvement [99], water-fertilizer control [100,101], tillage management [102], and adding or spraying exogenous substances, mainly silicon [103], and abscisic acid [104]); (2) bioremediation to reduce the Cd concentration in the soil or reducing the Cd content in rice grains through phyto-treatment [105] and microbiological treatments [106]; and (3) screening and breeding rice varieties with low Cd accumulation. However, agronomical measures and bioremediation are often faced with many problems such as the high investment cost, slow efficiency, high labor requirement, and difficulty preventing and controlling subsequent pollution. Therefore, screening and breeding varieties with low Cd accumulation are considered the most economical and effective way to reduce the risk of Cd pollution in rice.

### 5.1. Variety Selection and Cross-Breeding

The tolerance mechanisms of diverse rice genotypes under Cd stress differ; hence, the Cd absorption, transport, and accumulation greatly vary among rice genotypes. Comparing the Cd enrichment characteristics among the existing resources to select plant varieties with low Cd accumulation under different climatic conditions is the fastest method for safely utilizing Cd-contaminated soils. Currently, Zhao et al. screened the Cd content in the grains of 312 rice resources, among which 24 resources accumulated a Cd content within the national standard [107]. In addition, Jiang et al. established significant genotypic differences in Cd content accumulation in the grains of rice varieties using field tests in different regions of China including a series of low-Cd rice varieties such as Nanji 3, Guangliuzao, and Zhenong 992 [108]. Following increased Cd pollution in Hunan, a research group from the Institute of Subtropical Agriculture, the Chinese Academy of Sciences, in collaboration with other groups, conducted comparative studies on 110 rice varieties. They identified several rice varieties suitable for cultivation under different pollution types, pollution levels, and soils through pot experiments, field micro-plot trials, and field pilot tests [109].

For breeders, cross-breeding can transfer low-Cd-accumulation-related alleles to primal parents with high yield and disease resistance. Alternatively, the excellent alleles for controlling yield and disease resistance can be polymerized into low-Cd varieties to obtain low-Cd rice varieties with high performance. For example, Huang et al. [110] used the C5S sterile line of rice as the receptor parent and crossed it with Gumei 4 (containing *Pigm*), Tianjin wild rice (containing *Pi2-1*), and Mowanggu (containing *Pi49*) as the resistance source of rice blast. Meanwhile, the derivative line Cd14030 of *japonica* rice (Nipponbare) was used as the donor parent for the rice low-Cd accumulation gene *OsHMA3*. The material C5S (*Pi49*+*Pigm*+*OsHMA3*) with low Cd accumulation and resistance to rice blast was generated through gradual polymerization.

### 5.2. Mutation Breeding

Mutation breeding is a method that employs physical or chemical factors to induce a variation in the genetic characteristics of rice, followed by the selection of individual plants that vary from the offspring, which are then cultivated as new varieties. It has several advantages including a higher mutation frequency, requires a short time for stability using simple operations, and breaks the link between traits to achieve gene recombination [9]. For example, Ishikawa et al. obtained three *OsNRAMP5* loss-of-function mutants with a significantly lower Cd content in the roots and stems than the WT, and a Cd concentration in the grains lower than 0.05 mg·kg^−1^ using carbon ion-beam irradiation mutagenesis [111]. Meanwhile, Chen et al. [112] used a 12C^6+^ ion-beam to irradiate the 9311 rice variety to obtain a *T2-1* mutant with low Cd accumulation of about 28.6% lower than the parents.

Mutation breeding can also be combined with hybrid breeding. Based on hybrid recombination, the appropriate dose of mutagenesis treatment on unstabilized rice hybrids can increase the mutation frequency, expand the mutation spectrum, and break the gene linkage, which promotes the production of more mutation types during gene recombination. In rice, combining radiation mutagenesis with conventional hybridization technology yields higher mutation types and frequency than using either of the single treatments alone, which could produce better breeding outputs [113].

### 5.3. Marker-Assisted Breeding (MAS)

New rice varieties can be cultivated from mutants using molecular markers to trace the rice genetic composition, which shortens the breeding cycle, achieves precision breeding, improves breeding efficiency, and saves on screening costs. Four low-Cd-associated molecular markers have been developed to screen 5769 rice varieties using MAS technology to obtain 80 new lines carrying 1–3 low-Cd-associated molecular markers [114]. Field tests further revealed that some of the varieties among the 80 new lines developed had stable genetic characteristics of low-Cd after hybridization. Furthermore, a Tons molecular marker detection system based on a 2980 bp sequence (Tons) on chromosome 7 in the Luohong 4A rice variety with low Cd accumulation has been established [115]. Subsequently, a new low-Cd Tonys was selected in the Luohong 4A hybrids. At the same time, Ishikawa et al. confirmed *OsNRAMP5* as the gene responsible for the reduction in Cd absorption, based on which a MAS system used to screen varieties carrying *OsNRAMP5* in the mutant offspring population was constructed [111].

### 5.4. Genetic Engineering Breeding

More than 30 genes involved in Cd metabolism have been identified in rice; thus, low-Cd rice materials can be obtained by manipulating key genes regulating Cd accumulation through transgenic technology. For example, Shimo et al. inserted T-DNA into *OsLCD*, creating a new Cd-resistant mutant *lcd* through a screening experiment [53]. In addition, the Cd content in transgenic rice grains overexpressing *OsHMA2* was only half that of the WT [69]. Nevertheless, inhibiting the *OsLCT1* expression using RNAi technology reduced the Cd content in grains by 50%, with no impact on plant growth and the mineral content in the grains [51].

In addition, low-Cd rice has been obtained by a site-directed mutation on Cd-accumulation-related functional genes using the CRISPR/Cas9 gene-editing technology [116]. The CRISPR/Cas9 technology has also been used to induce targeted mutations on exons 1 and 3 of *OsLCT1* and exons 7 and 9 of *OsNRAMP5*, generating two *oslct1* mutants and two *osnramp5* mutants, respectively [117]. Evaluations of the Cd accumulation and agronomic traits of rice using pot and field experiments revealed that different amplitudes significantly reduced the Cd content in brown mutant rice. At the same time, low-Cd male sterile and restorer lines have been created by CRISPR using primal parents of hybrid rice as receptor materials. Hybridization has also generated new hybrid rice varieties with high yield, high quality, and low Cd accumulation [118]. For example, the *OsNRAMP5* gene was knocked-out in primal parents of two-line hybrid rice Huazhan and Longke 638S. Subsequently, the *osnramp5* mutant, Long-liang-you-huazhan was generated by a combination experiment. Further trials in heavily Cd-polluted fields revealed that the Cd content in brown rice was lower than 0.05 mg·kg^−1^, with no significant differences in yield, quality, and other agronomic traits observed compared to the WT [119].

## 6. Conclusions and Future Perspectives

Cd pollution is a serious environmental problem. Cd accumulation in the soil not only affects the growth and metabolism of rice, but also harms human health through the food chain. Therefore, understanding the transport mechanism of Cd and the factors influencing Cd accumulation in rice is crucial for developing effective Cd reduction strategies and preventing harm to the human body. In recent years, an increasing number of studies on the molecular mechanism of Cd metabolism in rice in China and other countries to clarify the physiological process and regulatory factors of Cd accumulation and transport from the soil to rice grains have been conducted, which lay an important theoretical foundation for future cultivation of low-Cd rice varieties [120]. Preliminary success has also been achieved in cultivating low-Cd rice by genetic engineering. However, the transporters that regulate Cd absorption and accumulation generally have a wide range of substrates. In addition to Cd, they also transport trace elements such as manganese, iron, and Zn, necessary for rice growth and development. Therefore, it is important during the improvement of the Cd resistance of rice to establish whether the knockout or overexpression of related genes has a negative impact on the acquirement of essential metal elements, which could inhibit rice growth or reduce rice yields. For example, the knockout of *OsNRAMP5* through gene editing not only reduced the Cd content in rice but also inhibited the absorption of other trace elements (especially manganese) [36,56,57,58]. This drawback limits its application in low manganese farmland, indicating the complexity and difficulty in improving metal transporters to reduce Cd absorption.

Future research on low-Cd rice should explore strategies to (1) enhance the comprehensive implementation of various agronomic measures to reduce the bioavailability of exogenous Cd and establish a safe and efficient cultivation technology system; (2) develop molecular markers closely linked to a low-Cd gene in rice, promote the application of molecular markers in breeding, and combine molecular breeding with conventional breeding; and (3) explore the molecular elements related to Cd metabolism in rice, implement polymerization breeding, and finally cultivate new low-Cd rice varieties with stable heredity, safety, and high quality.

## Figures and Tables

**Table 1:** Transporters and genes encoding transporters related to Cd accumulation in rice.

Gene Name	Gene ID	Main Expression Tissues	Subcellular Localization	Reference
*OsNRAMP1*	*LOC_Os07g15460*	Root tissues	Plasma membrane	[35]
*OsNRAMP5*	*LOC_Os07g15370*	Root epidermis, exodermis, lateral cortex and tissues around xylem in the vascular bundle	Plasma membrane	[36]
*OsZIP1*	*LOC_Os01g74110*	Root tissues	Plasma membrane	[37]
*OsZIP3*	*LOC_Os04g52310*	Parenchyma cells around enlarged vascular bundles in stem nodes	Plasma membrane	[38]
*OsZIP5*	*LOC_Os05g39560*	Root epidermis and parenchyma cells around xylem, parenchyma cells around xylem tissue in enlarged vascular bundle in stem nodes, parenchyma cells around xylem, and phloem of diffuse vascular bundles	Plasma membrane	[39]
*OsZIP6*	*LOC_Os05g07210*	Root and above-ground tissues	Plasma membrane	[40]
*OsZIP7*	*LOC_Os05g10940*	Parenchyma cells in root stelar, parenchyma cells around the enlarged vascular bundle in stem nodes	Plasma membrane	[41]
*OsZIP9*	*LOC_Os05g39540*	Root epidermis, parenchyma cells around the xylem in enlarged vascular bundle, parenchyma cells around xylem and phloem in diffuse vascular bundle in stem nodes	Plasma membrane	[39]
*OsIRT1*	*LOC_Os03g46470*	Epidermis and exodermis in the root elongation zone, cortex of mature root areas, companion cells in phloem of root stelar, phloem in stem nodes	Plasma membrane	[42,43]
*OsIRT2*	*LOC_Os03g46454*	Root tissues	Plasma membrane	[43]
*OsCd1*	*LOC_Os03g02380*	All root tissues	Plasma membrane	[44]
*OsHMA2*	*LOC_Os06g48720*	Root pericycle, phloem of enlarged vascular bundle, and diffuse vascular bundle in stem nodes	Plasma membrane	[45]
*OsHMA3*	*LOC_Os07g12900*	All root tissues	Tonoplast membrane	[46]
*OsABCG36* (*OsPDR9*)	*LOC_Os01g42380*	All root tissues except epidermis	Plasma membrane	[47]
*OsABCG43* (*OsPDR5*)	*LOC_Os07g33780*	Root and aboveground tissues	Plasma membrane	[48]
*OsFWL4*	*LOC_Os03g61440*	Root, flag leaf, and leaf sheath tissues	\	[49]
*CAL1*	*LOC_Os02g41904*	Root exodermis, parenchyma cells around xylem in the root and leaf sheath	\	[50]
*OsLCT1*	*LOC_Os06g38120*	Leaf, parenchyma cells around the enlarged vascular bundle and diffuse vascular bundle in Node I	Plasma membrane	[51]
*OsCCX2*	*LOC_Os03g45370*	Parenchyma cells in enlarged vascular bundles and diffuse vascular bundles in stem nodes	Plasma membrane	[52]
*OsLCD*	*LOC_Os01g72670*	Companion cells in phloem in the leaf, root vascular bundle	Cytoplasm, nucleus	[53]
*OsMTP1*	*LOC_Os05g03780*	Root and shoot tissues, leaf-specific sieve tube cells	Plasma membrane	[54]
*OsMTP2*	*LOC_Os01g62070*	Vascular bundles of stems and leaves, embryo sac wall of immature seeds	\	[55]

## Data Availability

Not applicable.

## Abbreviations

ABC	ATP-binding cassette transporter superfamily
CaCA	Cation/calcium superfamily
CAL1	Cadmium accumulation in leaf 1
CCXs	Cation/Ca exchangers
Cd	Cadmium
CDF	Cation diffusion facilitator
CKK	Cho Ko Koku
Cu	Copper
DVB	Diffuse vascular bundle
EVB	Enlarged vascular bundle
HMA	Heavy metal ATPase
Hsf	Heat shock transcription factor
lc5	Low cadmium 5
LCT	Low-affinity cation transporter
lncRNAs	Long non-coding RNAs
MAS	Marker-assisted breeding
MFS	Major facilitator superfamily
miRNAs	microRNAs
MPF	Membrane protein family
MTPs	Metal tolerance proteins
NRAMP	Natural resistance-associated macrophage protein
OsHB4	Homeodomain-containing protein 4
PCB	Parenchyma cell bridge
PSI	Photosystem I
PSII	Photosystem II
TVB	Transit vascular bundle
WT	Wild-type
Zn	Zinc
ZRTs	Zn-regulated transporters
